# Convolutional neural networks for the automatic segmentation of lumbar paraspinal muscles in people with low back pain

**DOI:** 10.1038/s41598-022-16710-5

**Published:** 2022-08-05

**Authors:** E. O. Wesselink, J. M. Elliott, M. W. Coppieters, M. J. Hancock, B. Cronin, A. Pool-Goudzwaard, K. A. Weber II

**Affiliations:** 1 Faculty of Behavioural and Movement Sciences, Amsterdam Movement Sciences, Vrije Universiteit Amsterdam (FGB), Van der Boechorststraat 9, 1081 BT Amsterdam, The Netherlands; 2 Faculty of Medicine and Health and the Northern Sydney Local Health District, The Kolling Institute, The University of Sydney, Sydney, Australia; 3 Department of Physical Therapy and Human Movement Sciences, Feinberg School of Medicine, Northwestern University, Chicago, IL USA; 4 Menzies Health Institute Queensland, Griffith University, Brisbane and Gold Coast, Australia; 5 Department of Health Professions, Faculty of Medicine, Health and Human Sciences, Macquarie University, Sydney, Australia; 6 SOMT University of Physiotherapy, Amersfoort, The Netherlands; 7 Division of Pain Medicine, Department of Anesthesiology, Perioperative and Pain Medicine, Stanford University, Palo Alto, CA USA

**Keywords:** Anatomy, Medical research, Mathematics and computing

## Abstract

The size, shape, and composition of paraspinal muscles have been widely reported in disorders of the cervical and lumbar spine. Measures of size, shape, and composition have required time-consuming and rater-dependent manual segmentation techniques. Convolutional neural networks (CNNs) provide alternate timesaving, state-of-the-art performance measures, which could realise clinical translation. Here we trained a CNN for the automatic segmentation of lumbar paraspinal muscles and determined the impact of CNN architecture and training choices on segmentation performance. T_2_-weighted MRI axial images from 76 participants (46 female; age (SD): 45.6 (12.8) years) with low back pain were used to train CNN models to segment the multifidus, erector spinae, and psoas major muscles (left and right segmented separately). Using cross-validation, we compared 2D and 3D CNNs with and without data augmentation. Segmentation accuracy was compared between the models using the Sørensen-Dice index as the primary outcome measure. The effect of increasing network depth on segmentation accuracy was also investigated. Each model showed high segmentation accuracy (Sørensen-Dice index ≥ 0.885) and excellent reliability (ICC_2,1_ ≥ 0.941). Overall, across all muscles, 2D models performed better than 3D models (*p* = 0.012), and training without data augmentation outperformed training with data augmentation (*p* < 0.001). The 2D model trained without data augmentation demonstrated the highest average segmentation accuracy. Increasing network depth did not improve accuracy (*p* = 0.771). All trained CNN models demonstrated high accuracy and excellent reliability for segmenting lumbar paraspinal muscles. CNNs can be used to efficiently and accurately extract measures of paraspinal muscle health from MRI.

## Introduction

Low back pain (LBP) is the leading cause of disability worldwide^1^ driven by a complex multifactorial inter-relationship between biological, psychological and social systems^2^. Various parameters of paraspinal muscle health (e.g., size, shape, and composition) have been acknowledged as potentially important biological markers in people with LBP^3^. However, the magnitude of, and association between, paraspinal muscle health and the clinical course of LBP remains largely unknown^4^. In some studies, a decrease in muscle volume or increase of fatty infiltration of the paraspinal muscles was highly associated with the presence and severity of LBP^5,6^, but other studies disagree^4^. Beyond differences between study samples, such conflicting results could be the consequence of differences in imaging and quantification methods to assess lumbar paraspinal muscle health^7,8^.

Paraspinal muscle morphometric and compositional measures are preferably quantified by magnetic resonance imaging (MRI) due to high soft tissue contrast^9^. However, quantitative musculoskeletal MRI measurements require manual segmentation of the muscle borders, which is time-consuming and user-dependent, representing a significant barrier to the translation of these quantitative MRI methods to clinical practice. Similar to other research fields (e.g., spinal cord injury and knee osteoarthritis)^10,11^, the development of time-efficient and fully-automated tissue segmentation techniques are needed to realise the potential implementation of (muscle) morphometric and compositional measures^12^ when clinically warranted.

Recent applications of deep learning methods, in particular convolutional neural networks (CNN), have shown potential for automating the segmentation of the cervical and lumbar paraspinal muscles from MRI^13,14^. CNNs are able to learn hierarchical spatial features^15^ with an increasing level of abstraction as the imaging inputs are processed through the network layers^16^. First, shallow layers collect low-level features (e.g., edges, contrasts) while deeper layers collect high-level features (e.g. shapes, localization) using filters whose receptive fields capture more global information^16^.

Weber et al. (2019) provided evidence that CNNs can be used to automate the calculation of both muscle volume and fat measures of the cervical spine extensor muscles with high segmentation performance (i.e., accuracy and reliability) using MR fat–water imaging^14^. Shen et al. (2021) demonstrated high performance of CNN for the segmentation of lumbar paraspinal muscles from T_2_-weighted axial images^13^. However, the latter focussed on the L4–L5 intervertebral disc level and thus volumetric measures of the paraspinal muscles traversing the entire lumbar spine were not available. Volumetric measures are clinically relevant, because the anatomical variability in muscle morphometry is dependent on segmental level in the axial profile^4^. Lumbar paraspinal muscle segmentation is a challenging task due to high anatomical variability within and between subjects^17^ and varying pixel intensity distributions within the muscles due to different levels of intramuscular fatty infiltration^4^ and B_0_ field inhomogeneity^18^. In addition, the estimates of the anatomical boundaries need to be accurate in lumbar paraspinal segmentation tasks because the allocation of false-positive voxels to the region of interest can possibly lead to inaccurate measures of muscle quality by including extramuscular tissue (e.g., bony tissue, extramuscular fatty infiltration).

Here, CNN models were trained to segment the entire volume of the lumbar paraspinal muscles. As modelling choices may influence CNN performance^19^, this technical report compared 2D and 3D CNN architectures with and without data augmentation. Furthermore, we investigated the importance of CNN network depth to understand the influence of high-level feature information on the segmentation of the paraspinal muscles. We believe the findings will provide insight into the relationship between CNN modelling choices and segmentation performance towards informing future efforts to optimize CNN segmentation frameworks and facilitate their implementation into clinical practice.

## Results

Using three-fold cross-validation, we randomly split the axial T_2_-weighted images of the lumbar spine (n = 76; 46 female; mean (SD) age: 45.6 (12.8) years; BMI: 26.9 (5.1)) into three training (n = 50) and testing datasets (N = 26). Descriptive statistics per training and testing fold are presented in Supplementary Table 1. Within each fold, we first trained the CNN models for 30,000 iterations using the training images. Then, we applied the trained model to the corresponding testing images. The trained CNN models segmented all axial slices in an image in 6.4 (0.1) seconds. Last, we evaluated the accuracy and reliability of the CNN segmentations across the folds compared to the manually segmented ground truth. Based on the ground truth segmentations, the mean (SD) muscle volumes of the erector spinae (left: 300.3 (75.6) ml; right: 294.7 (70.4) ml)) were larger than the psoas major (left: 157.3 (55.9) ml; right: 160.3 (55.7) ml) (*p* < 0.001), and the multifidus had the smallest muscle volumes (left: 120.6 (27.0) ml; right: 119.9 (25.7) ml) (*p* < 0.001).

### Interrater reliability (manual segmentation)

To compare the CNN model reliability to inter-human performance, we assessed the interrater reliability of manual segmentation between two raters in a subset of images (n = 25). Both raters had extensive training in lumbar spine anatomy and imaging^8^. High segmentation accuracy and excellent reliability were observed between the two manual raters (Sørensen-Dice index ≥ 0.904 and ICC_2,1_ ≥ 0.940, for all muscles).

### CNN accuracy and reliability

We assessed the segmentation accuracy using the Sørensen-Dice index as the primary outcome measure. The Jaccard index, conformity index, true positive rate, true negative rate, positive predictive value, and volume ratio were also calculated and are reported in Table 1. There was a high segmentation accuracy (Sørensen-Dice index ≥ 0.885) across the four CNN models for all muscles. Repeated-measures ANOVA showed significant main effects for model (*p* = 0.012), data augmentation (*p* < 0.001), and muscle (*p* < 0.001) and a significant model by muscle interaction (*p* < 0.001) (normality and sphericity assumed, *p* > 0.05). Overall, across all muscles, 2D models outperformed 3D models, and models trained without data augmentation outperformed models trained with data augmentation. The multifidus consistently had the lowest average CNN segmentation accuracy (Sørensen-Dice index 0.893–0.905) across all models.

**Table 1:** Performance of the CNN models. Data are presented as mean (SD).

	2D without DA	2D with DA	3D without DA	3D with DA
**Multifidus Left**				
Sørensen-Dice Index	**0.905 (0.021)**	0.902 (0.031)	0.897 (0.021)	0.893 (0.035)
Jaccard Index	**0.827 (0.034)**	0.823 (0.048)	0.815 (0.034)	0.805 (0.050)
Conformity Index	**0.789 (0.051)**	0.780 (0.085)	0.770 (0.053)	0.751 (0.103)
True Positive Rate	0.905 (0.032)	0.900 (0.045)	**0.907 (0.035)**	0.892 (0.052)
True Negative Rate	**0.999 (0.000)**	**0.999 (0.001)**	**0.999 (0.001)**	**0.999 (0.001)**
Positive Predictive Value	**0.907 (0.036)**	0.906 (0.038)	0.890 (0.043)	0.892 (0.041)
Volume Ratio	**1.001 (0.074)**	0.995 (0.064)	1.022 (0.075)	1.003 (0.082)
Volume ICC_2,1_ (95% CI)	**0.967 (0.949–0.979)**	0.962 (0.941–0.975)	0.954 (0.929–0.971)	0.941 (0.908–0.962)
**Multifidus Right**				
Sørensen-Dice Index	**0.900 (0.024)**	0.898 (0.028)	0.891 (0.021)	0.885 (0.032)
Jaccard Index	**0.819 (0.039)**	0.816 (0.044)	0.804 (0.034)	0.795 (0.043)
Conformity Index	**0.776 (0.061)**	0.770 (0.072)	0.754 (0.053)	0.738 (0.074)
True Positive Rate	0.900 (0.038)	**0.901 (0.040)**	0.895 (0.036)	0.888 (0.040)
True Negative Rate	**0.999 (0.001)**	**0.999 (0.001)**	**0.999 (0.001)**	**0.999 (0.001)**
Positive Predictive Value	**0.901 (0.041)**	0.897 (0.043)	0.890 (0.040)	0.885 (0.047)
Volume Ratio	**1.000 (0.062)**	1.007 (0.073)	1.009 (0.075)	1.007 (0.083)
Volume ICC_2,1_ (95% CI)	0.941 (0.910 –0.962)	0.948 (0.920–0.967)	**0.949 (0.922–0.967)**	**0.949 (0.922–0.967)**
**Erector Spinae Left**				
Sørensen-Dice Index	**0.931 (0.020)**	0.924 (0.026)	0.925 (0.019)	0.918 (0.029)
Jaccard Index	**0.871 (0.034)**	0.860 (0.043)	0.862 (0.032)	0.848 (0.039)
Conformity Index	**0.851 (0.048)**	0.834 (0.069)	0.838 (0.046)	0.818 (0.067)
True Positive Rate	**0.934 (0.034)**	0.921 (0.039)	0.930 (0.026)	0.927 (0.038)
True Negative Rate	**0.998 (0.001)**	**0.998 (0.002)**	0.997 (0.001)	0.997 (0.001)
Positive Predictive Value	**0.930 (0.030)**	0.929 (0.036)	0.922 (0.034)	0.909 (0.034)
Volume Ratio	**1.005 (0.056)**	0.993 (0.063)	1.010 (0.053)	1.021 (0.061)
Volume ICC_2,1_ (95% CI)	0.973 (0.958–0.982)	0.964 (0.945–0.977)	**0.980 (0.968–0.987)**	0.969 (0.949–0.981)
**Erector Spinae Right**				
Sørensen-Dice Index	**0.928 (0.022)**	0.923 (0.028)	0.920 (0.019)	0.916 (0.036)
Jaccard Index	**0.867 (0.037)**	0.858 (0.047)	0.853 (0.033)	0.843 (0.050)
Conformity Index	**0.844 (0.054)**	0.830 (0.069)	0.826 (0.047)	0.809 (0.097)
True Positive Rate	**0.928 (0.033)**	0.927 (0.035)	0.920 (0.032)	0.926 (0.049)
True Negative Rate	0.997 (0.002)	0.997 (0.003)	**0.998 (0.001)**	0.997 (0.002)
Positive Predictive Value	**0.930 (0.033)**	0.921 (0.050)	0.922 (0.031)	0.905 (0.037)
Volume Ratio	**0.999 (0.059)**	1.011 (0.082)	**0.999 (0.057)**	1.025 (0.071)
Volume ICC_2,1_ (95% CI)	0.970 (0.954–0.981)	0.939 (0.906–0.961)	**0.973 (0.958–0.983)**	0.954 (0.926–0.972)
**Psoas Major Left**				
Sørensen-Dice Index	0.929 (0.020)	0.915 (0.041)	**0.930 (0.020)**	0.927 (0.024)
Jaccard Index	0.868 (0.034)	0.846 (0.066)	**0.870 (0.033)**	0.858 (0.045)
Conformity Index	0.846 (0.048)	0.810 (0.108)	**0.848 (0.045)**	0.831 (0.073)
True Positive Rate	0.934 (0.027)	0.904 (0.071)	0.938 (0.027)	**0.939 (0.025)**
True Negative Rate	**0.999 (0.000)**	**0.999 (0.001)**	**0.999 (0.000)**	**0.999 (0.001)**
Positive Predictive Value	0.925 (0.034)	**0.931 (0.033)**	0.923 (0.035)	0.910 (0.052)
Volume Ratio	**1.012 (0.055)**	0.973 (0.090)	1.018 (0.057)	1.036 (0.082)
Volume ICC_2,1_ (95% CI)	**0.990 (0.984–0.994)**	0.954 (0.924–0.971)	0.981–0.967–0.989)	0.981 (0.967–0.989)
**Psoas Major Right**				
Sørensen-Dice Index	**0.932 (0.019)**	0.921 (0.032)	**0.932 (0.020)**	0.923 (0.051)
Jaccard Index	**0.874 (0.033)**	0.854 (0.053)	0.873 (0.034)	0.856 (0.057)
Conformity Index	**0.853 (0.045)**	0.825 (0.079)	0.852 (0.047)	0.824 (0.106)
True Positive Rate	**0.938 (0.029)**	0.930 (0.036)	0.937 (0.031)	0.920 (0.059)
True Negative Rate	**0.999 (0.001)**	0.998 (0.001)	**0.999 (0.001)**	**0.999 (0.001)**
Positive Predictive Value	**0.928 (0.036)**	0.913 (0.052)	**0.928 (0.037)**	0.927 (0.043)
Volume Ratio	1.012 (0.059)	1.023 (0.079)	1.013 (0.062)	**0.996 (0.090)**
Volume ICC_2,1_ (95% CI)	**0.985 (0.977–0.990)**	0.974 (0.960–0.984)	0.984 (0.975–0.990)	0.969 (0.952–0.980)

*DA*  Data augmentation. Bold = Highest measure across all models.

**Figure 1:**
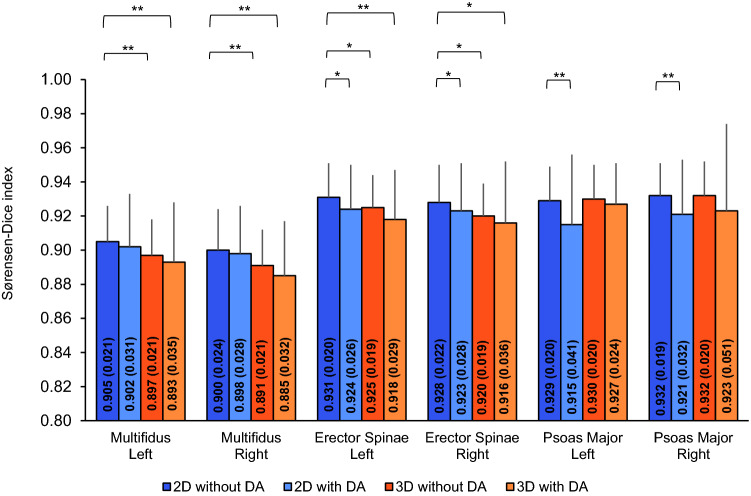
Performance of the CNN models: Sørensen-Dice index (primary outcome). Data are presented as mean and the error bars represent 1SD. Significance levels are presented for the model with the highest CNN segmentation accuracy (2D model without data augmentation) compared to the other models for all muscles. *DA*  Data augmentation, *p ≤ 0.05, **p ≤ 0.001.

Next, we performed post-hoc paired sample t-tests to compare the performance of the 2D model trained without data augmentation to the other models on a muscle-by-muscle basis. For both the left and right multifidus, the 2D model without data augmentation had the highest segmentation accuracy of the CNN models with the accuracy being significantly higher than the 3D models trained with and without data augmentation (*p* ≤ 0.001) and had similar segmentation accuracy to the 2D model with data augmentation (*p* > 0.214) (Fig. 1). For both the left and right erector spinae, the 2D model without data augmentation also had highest segmentation accuracy of the CNN models with the difference in accuracy being significantly higher than the 2D model trained with data augmentation (*p* ≤ 0.033) and the 3D models trained with without data augmentation (*p* ≤ 0.020 and *p* ≤ 0.003, respectively). For both the left and right psoas major, the 2D model trained without data augmentation had similar segmentation accuracy to the 3D models trained with and without data augmentation (*p* ≥ 0.524 and *p* ≥ 0.109, respectively). The difference in accuracy for the 2D model trained without data augmentation was significantly higher than the 2D model trained with data augmentation (*p* < 0.001).

The 2D model without data augmentation had volume ratios close to 1.000 for all muscles (0.999–1.012) with mean differences in muscle volumes for the left multifidus −0.55 (7.4) ml), right multifidus −0.57 (9.7) ml, left erector spinae 1.85 (19.12) ml), right erector spinae −0.16 (19.3) ml, left psoas major 0.86 (8.0) ml, and right psoas major −0.67 (9.5) ml.

**Figure 2:**
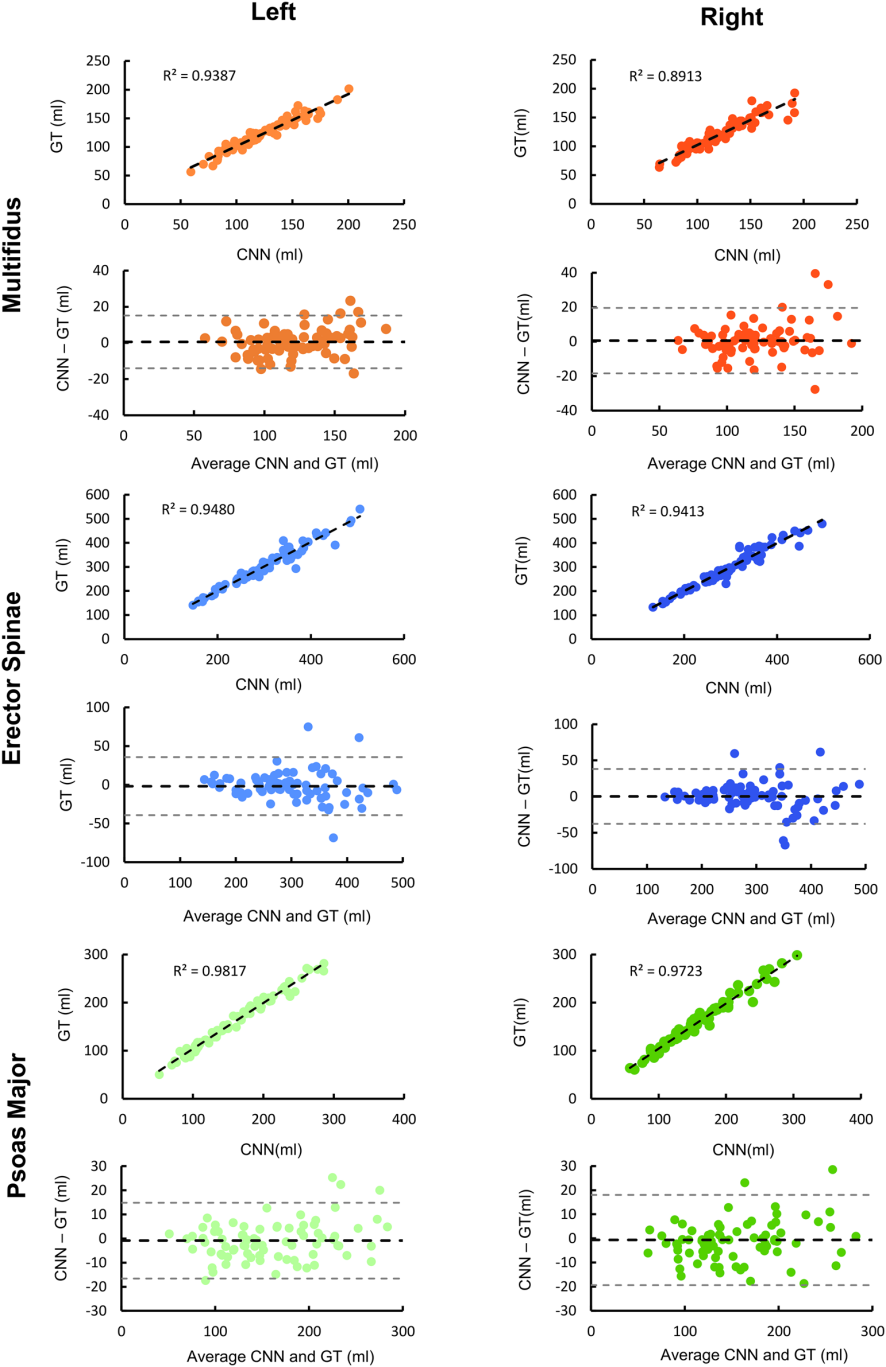
Reliability and accuracy of the 2D CNN model trained without data augmentation. Bland Altman (black dashed line = mean error, grey dashed lines = 95% limits of agreement) and correlation plots (black dashed line = best fit line) are shown for the volumes (ml) of the left and right paraspinal muscles. *GT*  Ground truth.

**Figure 3:**
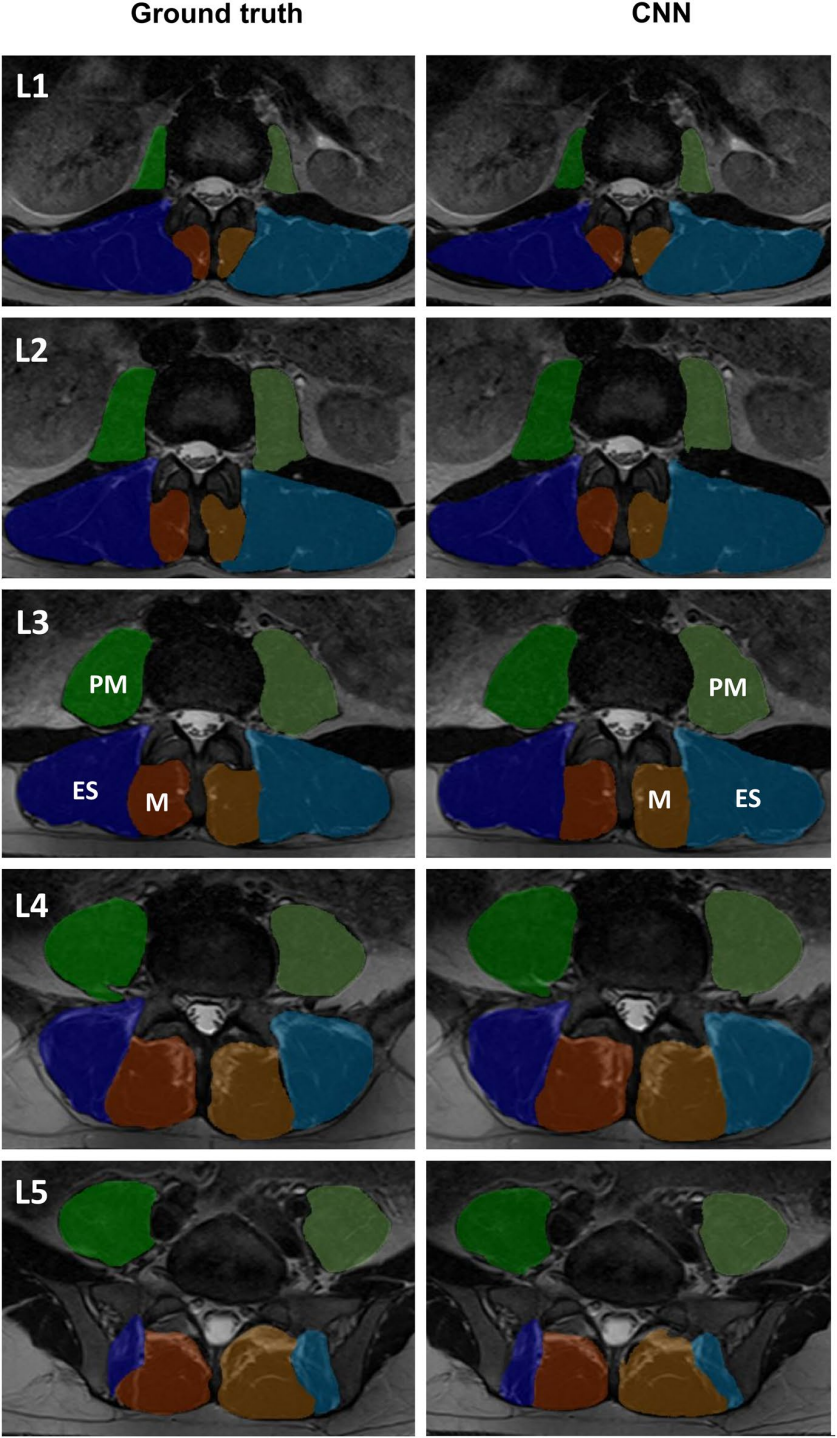
2D renderings at the L1–L5 vertebral levels with the paraspinal muscle segmentations superimposed from the ground truth and 2D CNN model trained without data augmentation. CNN masks of the right multifidus (dark orange), left multifidus (light orange), right erector spinae (dark blue), left erector spinae (light blue), right psoas major (dark green), left psoas major (light green) are shown. *ES*  Erector spinae, *M*  Multifidus, *PM*  Psoas Major.

**Figure 4:**
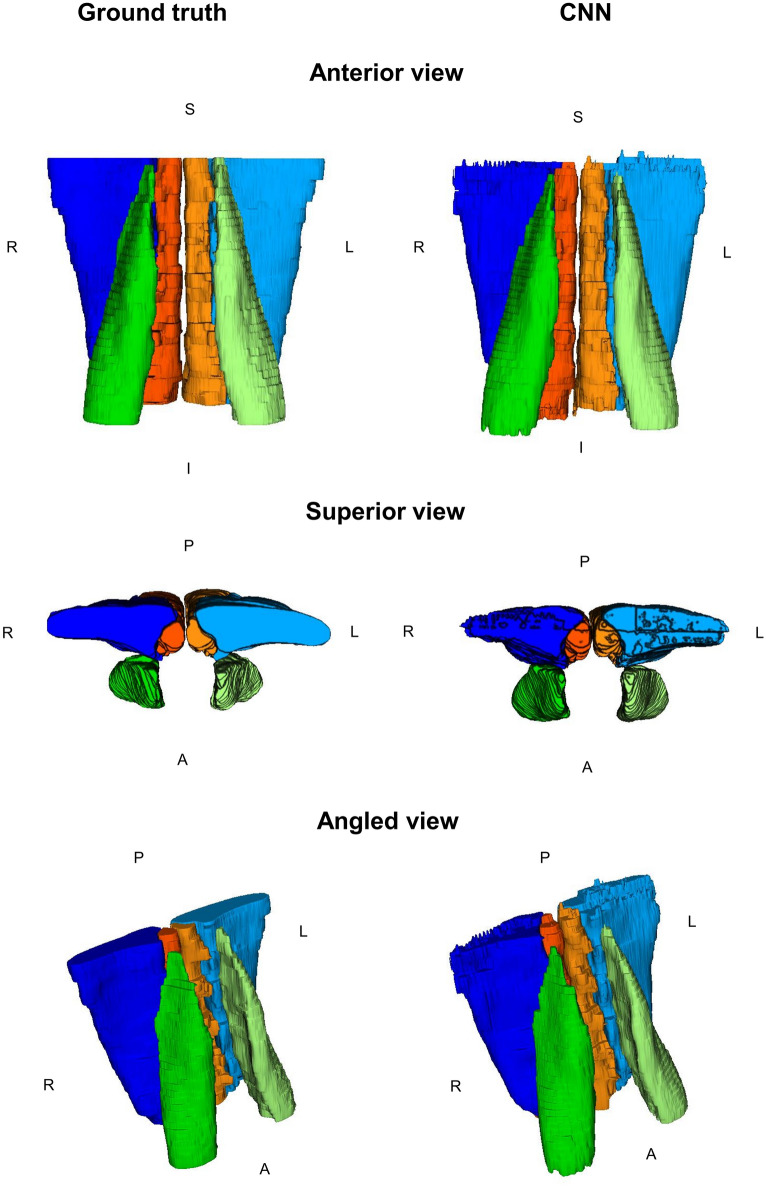
3D renderings of the paraspinal muscle segmentations from the 2D CNN model trained without data augmentation. CNN masks of the right multifidus (dark orange), left multifidus (light orange), right erector spinae (dark blue), left erector spinae (light blue), right psoas major (dark green), left psoas major (light green) are shown. *R*  Right, *L*  Left, *S*  Superior, *I*  Inferior, *A*  Anterior, *P*  Posterior.

Reliability between the CNN muscle volume measures with respect to the ground truth was measured using intraclass correlation coefficients (ICC_2,1_) (Table 1). Reliability was excellent (ICC_2,1_ ≥ 0.941) across the four CNN models for all muscles. For the 2D model trained without data augmentation, the left and right psoas major had the highest reliability (ICC_2,1_ ≥ 0.985). Reliability, accuracy and example renderings of the segmentations from the 2D model trained without data augmentation are presented in Figs. 2, 3 and 4.

### Depth of CNN network

CNN network depth increases the level of abstraction by extracting high-level features capturing broad based information, such as localization, coarse spatial grid information (e.g., shapes), and relationships between tissues on a global scale^20^. Therefore, we retrained the 2D model without data augmentation using a deeper U-Net model with an extra network layer of 512 filters to investigate the influence of CNN network depth and high-level feature information on the segmentation of paraspinal muscles. Increasing the CNN depth did not significantly improve the segmentation accuracy (repeated measures ANOVA with factors of model depth and muscle, *p* = 0.771).

## Discussion

Four CNN models (2D and 3D models with and without data augmentation) were trained and tested for automatic segmentation of the lumbar paraspinal muscles from axial T_2_-weighted images. All models were trained using a modified U-Net architecture designed for image segmentation. Overall, CNN segmentation accuracy was high, and the reliability was excellent for each model compared to the ground truth (Sørensen-Dice index ≥ 0.885, ICC_2,1_ ≥ 0.941). Furthermore, we provide evidence for higher performance using 2D compared to 3D models and higher performance for models trained without data augmentation versus with data augmentation. The 2D model trained without data augmentation demonstrated the highest average CNN segmentation accuracy across the muscles (Sørensen-Dice index ≥ 0.900, ICC_2,1_ ≥ 0.941).

Compared to Shen et al. (2021), we demonstrated improved outcomes for the erector spinae but slightly inferior performance for the multifidus and psoas major^13^. Their model, however, was limited to one axial slice (L4-L5 intervertebral disc level). In contrast, our approach included the entire superior-inferior expanse of the lumbar paraspinal muscles (L1-L5 vertebral levels) allowing us to capture 3D information of muscle morphometry, which provides a more complex and complete representation of the lumbar spine anatomy.

In agreement with Desai et al. (2019), we provide further evidence for higher performance of 2D (M × N × 1) over 3D (M × N × 32) models^19^. The 3D models in general had higher volume ratios, lower conformity index, and lower positive predictive values compared to the 2D models, which suggests the 3D models were likely including more false-positive voxels leading to larger segmentations. The lower performance of the 3D models may be partially explained by the anisotropic resolution of the images with a slice thickness of 5.0 mm and an in-plane resolution of 0.39 × 0.39 mm^2^, and 3D segmentation models have been shown to perform suboptimally when slice resolution is much larger than the between-slice resolution^21^. In fact, relatively small isotropic (3 × 3 × 3) 3D convolutional filter sizes may not be able to learn useful features from anisotropic voxels due to the varying information density along each dimension^22^.

Next, in 3D models, the stochastic approximation of the gradient of the loss function is updated on larger inputs (i.e., the gradient of the loss function is updated on lower number of model parameters per voxel, relatively)^23^. As such, the 3D networks could provide less specific and accurate gradient calculations.

Finally, 3D segmentation models use more computationally complex convolutions, allowing depth-wise features (along the z-axis) to be extracted throughout the network, resulting in a higher GPU memory footprint^10,24^. We chose a batch size to approximate maximal computational capacity and equivalent GPU memory footprint between models. As such, we used a limited batch size (n = 10, number of samples = 1) for our 3D models which may have led to less stable feature regularization compared to our 2D models (n = 50, number of samples = 4)^19^. Regularization is a technique to reduce the generalization error by including a penalty term to prevent the model of overfitting to the training data as a result of complex co-adaptations of model units^25^. Batch normalization is most commonly used in deep learning for regularization, but appears to lead to inaccurate batch estimation and higher model error in smaller batch sizes^26^. Hence, we used instance normalization (i.e., normalizing the feature maps per image) for all models to ensure that the contrast is not skewed by batched images with different input image contrast ranges^26,27^. However, CNN performance between different normalization techniques across different batch sizes in training CNNs for the segmentation of paraspinal muscles remains unclear. More research should be conducted to investigate CNN paraspinal muscle segmentation performance across different normalization techniques and batch sizes.

If memory costs for volumetric 3D convolutions can be reduced, the CNN performance for 3D models is likely to improve as optimizing 3D training parameters will be less restricted by total GPU memory. Several options have been suggested to reduce GPU memory costs for volumetric 3D convolutions. First, 2.5D (M × N × *t*) convolutions have been suggested to include volumetric information without the increase in network size^28^. The 2.5D network uses a stack of *t* continuous 2D slices across different orthogonal planes to segment the central slice. However, 2.5D networks may also perform suboptimally in anisotropic imaging datasets^28^. As such, more research needs to be conducted to investigate the validity of 2.5D networks for the segmentation of paraspinal muscles in anisotropic datasets. Second, implementing automatic mixed precision (AMP) training offers significant computational speedup and lowers GPU memory footprints by performing the operation in half-precision (float16) format and storing minimal information in single-precision (float32) in critical parts of the network^29^. AMP has been shown to be effective for reducing the GPU memory footprint and efficiency of CNN training while maintaining model accuracy^29^. Future research needs to be conducted to optimize the trade-off point between performance and computational costs in CNN training on biomedical volumes of the paraspinal muscles.

While data augmentation has been used to increase network expressivity, we provide further evidence it may reduce precision in a homogenous dataset with a standardized imaging acquisition protocol, similar to findings reported elsewhere^19^. In other words, due to equivalent imaging and clinical parameters between our training and testing dataset, data augmentation could result in an overestimation of imaging and anatomical heterogeneity. More research should be conducted to investigate optimal data augmentation parameters in more heterogenous imaging datasets where data augmentation may improve model performance and generalizability.

Improved performance was not realized with a deeper CNN network architecture (one extra network layer with 512 filters). It remains questionable if deeper and more complex networks are specifically needed for paraspinal muscle segmentation. Other recent work on training CNNs on thigh muscle volumes showed that deeper networks with short-cut connections and variety of convolutional block structures only led to marginal CNN improvements^30^. One explanation is that high-level information captured with deep layers of the network may not contribute as much to the results as the low-level image features, such as edges and contrast^30^. Future research to optimize the trade-off point between network depth, number of filters per layer, and segmentation performance in training 2D and 3D CNN models for paraspinal muscle segmentation is needed.

There was a significant main effect for muscles, with the multifidus having the lowest average CNN segmentation accuracy across all models. This difference can be explained by the relative magnitude of downsampling within the network with respect to the total volume of the muscles^20^. Compared to the erector spinae and psoas major, the multifidus has significantly smaller muscle volume. Hence, the multifidus could be exposed to more feature loss of spatial context information in the deeper layers, where the receptive fields of the filters compromises more high-level features^20^. Furthermore, the multifidus appears to have high anatomical variability between and within participants^17^. As such, it is more challenging for a CNN to learn the delineation of the multifidus compared to the erector spinae and psoas major across the entire expanse of the lumbar spine. Future work will focus on developing different CNN models to improve the segmentation accuracy for muscles with relatively small region of interest and high anatomical variability.

The CNN with the highest segmentation performance across all muscles (2D without data augmentation) reached human-level performance and was highly time-efficient. While not used in this study, post-processing transformations (through spatial connection and closing analysis) can reduce false-positive classified voxels by retaining the largest dense connected 3D-volume for each muscle^30^. These transformations can be helpful for clinical implementation to further improve CNN accuracy.

We provide promising results that CNNs can be used to automatically extract accurate measures of paraspinal muscle volume. As such, CNNs can improve the translation of warranted MRI methods to quantify paraspinal muscle health in clinical practice and large cohort studies. However, paraspinal muscles health compromises more than muscle size and shape and can also be characterized by the magnitude of intramuscular fatty infiltration, with more fatty infiltration being a sign of poor muscle health^31^. In T_2_-weighted images, fully-automated thresholding methods can be applied within the muscles to transform the image into regions of fat and muscle^32^. By automating muscle segmentation, the CNN can reduce the time and rater-dependency in calculating muscle fat infiltration, providing another measure of muscle health to complement measures of muscle size and shape. Future work will focus on developing CNN methods that generalize across different sites, sequence parameters, and image contrasts to develop quantitative measures of muscle health, controlling for sex as a biological variable, age, as well as race and ethnicity.

## Limitations

While we explored 2D versus 3D CNN models, other hyperparameters could influence the CNN performance^19^, and were not investigated in this study (e.g., loss function, learning rate, batch size, optimizer, etc.). Optimizing these parameters would likely improve segmentation performance further. Second, the use of images acquired from the same center and scanner, with equivalent sequences and parameters, may reduce the generalizability of our findings for more heterogenous datasets^14^. In large multi-site datasets with diverse spinal pathology (e.g., scoliosis, spondylolisthesis, spondyloarthropathies etc.) data augmentation may have benefit. Third, training and testing was limited to the manual segmentations of a single rater^13,14^. However, this concern is mitigated due to the excellent interrater reliability between the two raters (ICC_2,1_ ≥ 0.940) with negligible differences in muscle volume for the multifidus, erector spinae, and psoas major muscles.

## Conclusion

All trained CNN models demonstrated high segmentation performance and excellent reliability for segmenting lumbar paraspinal muscles, with peak CNN performance using a 2D model trained without data augmentation. The minimal time required to segment lumbar paraspinal muscles using CNN models enables the efficient quantification of large datasets. The findings provide insight in the relationship between CNN modelling choices and segmentation performance and can inform future efforts towards optimizing CNN segmentation frameworks and facilitating their implementation into clinical practice.

## Methods

### Participants

MRI scans from 76 participants (46 female; mean (SD) age: 45.6 (12.8) years; BMI: 26.9 (5.1)) were obtained from a prospective observational longitudinal study, exploring risk factors for recurrence of LBP^33^. Inclusion criteria were recovery from a previous episode of acute non-specific LBP within the last 3 months. Exclusion criteria were previous spinal surgery, contraindications to MRI, and inability to complete primary follow-up electronically. All applicable institutional and governmental regulations concerning the ethical use of human volunteers were followed during the course of this research according to the Declaration of Helsinki. Prior to working with the dataset, all personal identifying information was removed, and all participants provided written informed consent. The study was approved by the Macquarie Human Ethics Committee (Ref no: 5201200547)^33^.

### Image acquisition and processing

Lumbar spine T_2_-weighted axial images were acquired on a 3.0 Tesla General Electric MR Scanner (Milwaukee, WI, USA) with a spin-echo sequence (TR = 5 ms, TE = 0.116 ms, slice thickness = 4 mm, flip angle = 120°, pixel bandwidth = 219 Hz). Two blinded, independent raters with extensive training in lumbar spine anatomy and imaging manually segmented the muscles of interest (i.e., left and right multifidus, erector spinae, and psoas major) using anatomical cross-references as previously described^8^. Manual segmentation took 35.6 (5.8) minutes per person. One rater (EOW) segmented the entire dataset (n = 76), which was used as the ground truth for training and testing the CNN. The other rater (CB) independently segmented a subset of the dataset (n = 25) to assess the interrater reliability of manual segmentation. The images were randomly split into a three different training (n = 50) and testing dataset folds (N = 26) using k-fold cross-validation (*k* = 3). K-fold cross-validation is an internal model validation, where models are trained multiple times with different training and testing datasets to generate more generalizable models and to correct for the stochasticity of CNN learning^34^.

At the pre-processing phase, first all images were resampled to a consistent voxel size (0.39 mm × 0.39 mm × 5 mm). Then, the range of pixel values were normalized per subject to improve field homogeneity of the images. After the pre-processing phase, the images were cached to the GPU, or smart-cached to the RAM (choice is dependent on total memory costs), to reduce I/O costs and improve training speed. Data augmentation, model training, and model testing were performed using MONAI, an open-source community supported, Pytorch-based framework for deep learning in healthcare imaging^35^.

### Modified U-Net architecture

**Figure 5:**
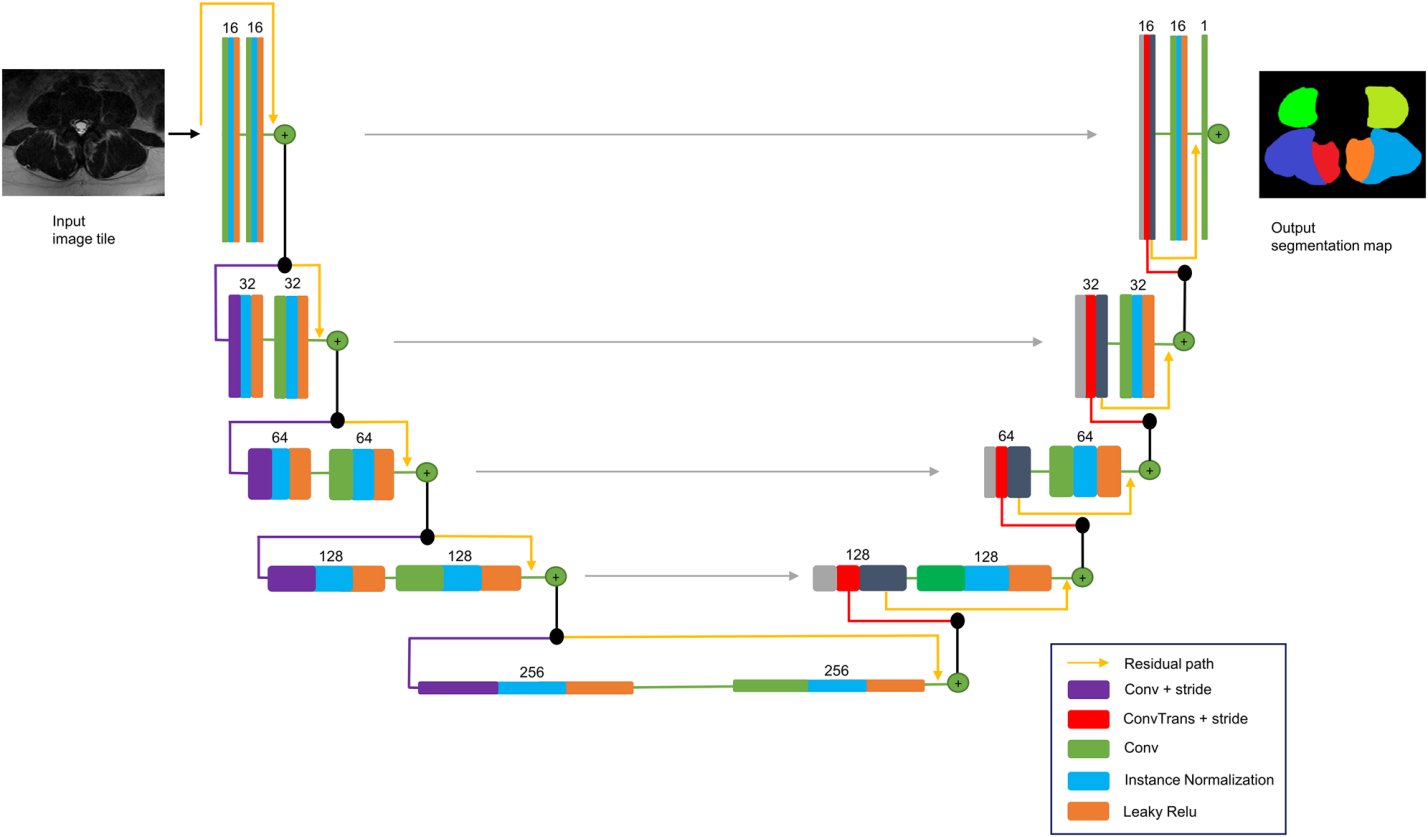
Network topology of the modified U-Net architecture.

We used a modified U-Net architecture for image segmentation (Fig. 5). U-Net is the state of the art CNN architecture, primarily designed for image segmentation^15^. The basic structure of a U-Net consist of an encoder and decoder synthesis path with multiple resolution steps^36^. Each level in our encoder path contains two 3 × 3 (× 3 in 3D) convolutions, an instance normalization layer followed by a Leaky Rectified Linear Unit (Leaky Relu)^37,38^. In contrast to the conventional U-Net architecture, the pooling layer^39^ for downsampling was replaced by a convolutional layer with a stride of 2 for downsampling as proposed by Kerfoot et al. (2019)^37^. This optimizes CNN learning efficiency through downsample operations while also reducing the number of layers in the network units^37^. At the first convolutional layer, a stride of 1 was used to prevent immediate downsampling of the input image. In the decoder of the synthesis path, transpose convolutions with stride of 2 were used for up-convolutions. Skip-connections are used to concatenate feature maps from the encoder to those of the same resolution in the decoder^15^. At each stage, a residual learning framework is implemented by adding the input of each stage to the output of its last convolutional layer (Fig. 5). This framework has been used to avoid degradation of CNN performance caused by diminishing gradients in the weight vector^40^.

### Training

The models were trained on a NVIDIA RTX 3070 24 GB graphical processing unit (GPU, NVIDIA, Santa Clara, CA) (optimizer = AdamW; loss function = DiceCEloss; weight decay = 0.0001; learning rate = 0.001). The Adam optimizer with decoupled weight decay (AdamW) was used because of better generalization to a testing dataset than the conventional ADAM with ℓ2 regularization^41^. In this approach, the weight decay was decoupled from the optimization steps with respect to the loss function, because the combination of adaptive gradients and ℓ2 regularization appears to lead to larger gradient amplitudes being regularized compared to weight decay specifically^41^. The images were randomly cropped to a spatial window size with the center being a foreground or background voxel based on a positive/negative ratio of one. The spatial window size was reduced to 50% of the field-of-view along superior-inferior axis in the 3D models (i.e., 256 × 256 × 32). The 2D models were trained slice-wice using individual axial slices with a spatial window size of 256 × 256 × 1. Before training, the unitary dimension (M × N × 1) was squeezed to generate true 2D patches. We chose a batch size to approximate maximal computational capacity with equivalent GPU memory footprint between models. Batch sizes of 10 (number of samples = 1) and 50 (number of samples = 4) were used for the 3D and 2D models, respectively. All models were initialized with random weights using equivalent randomizations, and the deterministic seed was set to zero. The model with the highest average segmentation accuracy was retrained on all three training folds and compared with a deeper U-Net model by including one extra layer with 512 filters, to investigate the clinical importance of CNN network depth and high-level feature information for the segmentation of paraspinal muscles.

### Data augmentation

The training dataset was augmented to increase the variability in the training images^15^. An augmented dataset of 1000 images was generated by applying a series of random affine spatial transformations, including scaling (−2.5–2.5%), mirroring along the left–right axis, rotation (x = *−*2.5–2.5°, y = −2.5–2.5°, z = −2.5–2.5°) and translation (in voxels relative to the centre of the input image, x = −25–25 voxels, y = −25–25 voxels, z = −2–2 voxels). These specific augmentation hyperparameters were chosen to mimic variations in positioning on the scanner bed and to prevent the network from fixating on specific regions of its perceptive field^37,42^. Furthermore, elastic deformations (sigma range = 6–8, magnitude range = 50–100, padding = ‘border’) were used to add more geometrical variability to the morphometric properties of the paraspinal muscles and increase the model generalisability to unseen datasets^15^.

### Evaluation of CNN segmentation performance

CNN segmentation accuracy was measured using the Sørensen-Dice index as the primary outcome and the Jaccard index, conformity coefficient, true positive rate, true negative rate, positive predictive value, and volume ratio as secondary outcomes (Table 2). CNN segmentation accuracy between models and muscles was compared for the primary outcome using repeated-measures ANOVA with factors of model, data augmentation, and muscle and all interactions (i.e., full factorial). Post-hoc paired sample t-tests were used to compare the model with the highest performance to all other models (α = 0.05). Furthermore, repeated-measures ANOVA with factors of model and muscle and a model by muscle interaction was used to compare CNN segmentation accuracy between the model with the highest performance to a deeper U-Net model. Residuals between models were tested for normality and sphericity using Skewness, Kurtosis, Shapiro–Wilk, Q–Q plots, and Mauchly’s test of Sphericity. Interrater reliability for the ground truth was measured using intraclass correlation coefficient (ICC_2,1_) between two manual raters. The reliability of the CNN model with the highest performance was assessed against the ground truth for muscle volume (ml) using ICC_2,1_, Bland–Altman plots, and correlation plots. All statistical analyses were performed using SPSS (IBM SPSS Statistics for Windows, version 26, IBM Corp., Armonk, N.Y., USA).

**Table 2:** Segmentation metrics.

Metric	Equation	Range	Meaning
S*ø*rensen-Dice Index	$\frac{2\times \left|\hbox{SM}\cap \hbox{GT}\right|}{|\hbox{SM}| + |\hbox{GT}|}$	0–1	Spatial overlap between masks
Jaccard Index	$\frac{|\hbox{SM}\cap \hbox{GT}|}{|\hbox{SM}| + |\hbox{GT}| - |\hbox{SM }\cap \hbox{GT}|}$	0–1	Spatial overlap between masks
Conformity Coefficient	$1- \frac{\hbox{FP }+\hbox{FN}}{\hbox{TP}}$	< 1	Ratio of incorrectly and correctly segmented voxels
True Positive Rate (TPR)	$\frac{\hbox{TP}}{\hbox{TP }+\hbox{FN}}$	0–1	Sensitivity
True Negative Rate (TNR)	$\frac{\hbox{TN}}{\hbox{TN }+\hbox{FP}}$	0–1	Specificity
Positive Predictive Value (PPV)	$\frac{\hbox{TP}}{\hbox{TP }+\hbox{FP}}$	0–1	Precision
Volume Ratio	$\frac{\hbox{SM}}{\hbox{GT}}$	≥ 0	Ratio of mask volumes

*SM*  segmentation mask, *GT*  ground truth mask, *TP*  true positive (i.e., voxels correctly segmented as muscle), *TN*  true negative (i.e., voxels correctly segmented as background), *FP*  false positive (i.e., voxels incorrectly segmented as muscle), *FN*  false negative (i.e., voxels incorrectly segmented as background).

## Supplementary Information

Supplementary Information.

## Data Availability

The de-identified datasets used in this study are available from the corresponding author upon reasonable request.
