# Supplementary material for: Convolutional neural networks for the automatic segmentation of lumbar paraspinal muscles in people with low back pain
Source: Sci Rep. 2022 Aug 5;12:13485. doi: 10.1038/s41598-022-16710-5 (PMC9355981; doi:10.1038/s41598-022-16710-5)
Supplement: Supplementary file 1 — Supplementary Information. [file 41598_2022_16710_MOESM1_ESM.docx]

**Supplementary Table 1:** Descriptive statistics for each training and testing fold.

|  | | **N** | **Mean** | **Standard Deviation** |
| --- | --- | --- | --- | --- |
| Age | Fold 1 training dataset | 50 | 45.0 | 12.9 |
|  | Fold 1 testing dataset | 26 | 46.7 | 12.9 |
|  | Fold 2 training dataset | 50 | 46.9 | 13.2 |
|  | Fold 2 testing dataset | 26 | 43.0 | 11.9 |
|  | Fold 3 training dataset | 50 | 44.3 | 12.3 |
|  | Fold 3 testing dataset | 26 | 48.0 | 13.6 |
|  |  | **N** | **Mean** | **Standard Deviation** |
| BMI | Fold 1 training dataset | 50 | 26.9 | 5.5 |
|  | Fold 1 testing dataset | 26 | 26.9 | 4.2 |
|  | Fold 2 training dataset | 50 | 27.4 | 4.9 |
|  | Fold 2 testing dataset | 26 | 26.1 | 5.4 |
|  | Fold 3 training dataset | 50 | 26.4 | 4.9 |
|  | Fold 3 testing dataset | 26 | 27.9 | 5.4 |
|  |  | **N** | **% female** | |
| Sex | Fold 1 training dataset | 50 | 58.0 | |
|  | Fold 1 testing dataset | 26 | 65.4 | |
|  | Fold 2 training dataset | 50 | 64.0 | |
|  | Fold 2 testing dataset | 26 | 53.8 | |
|  | Fold 3 training dataset | 50 | 60.0 | |
|  | Fold 3 testing dataset | 26 | 61.5 | |
